# Weiszfeld, tree-seed, and whale optimization algorithms comparison via locating transportation facilities with weightings considering the vulnerability and uncertainty

**DOI:** 10.1371/journal.pone.0269808

**Published:** 2022-06-14

**Authors:** Emre Demir

**Affiliations:** Department of Civil Engineering, Faculty of Engineering and Natural Sciences, Antalya Bilim University, Antalya, Turkey; Eastern Mediterranean University: Dogu Akdeniz Universitesi, TURKEY

## Abstract

Searching for an optimum transportation facility location with emergency equipment and staff is essential for a specific region or a country. In this direction, this study addresses the following problems. First, the performances of the Weiszfeld, tree–seed, and whale optimization algorithms are compared, which is the first of its kind in the literature. Second, a new approach that tests the importance parameters’ effectiveness in searching for an optimum transportation facility location with emergency equipment and staff is proposed. The Weiszfeld algorithm finds viable solutions with compact data, but it may not handle big data. In contrast, the flexibility of the tree–seed and whale optimization algorithm is literally an advantage when the number of parameters and variables increases. Therefore, there is a notable need to directly compare those algorithms’ performances. If we do, the significance of extending the number of parameters with multiple weightings is appraised. According to the results, the Weiszfeld algorithm can be an almost flexible technique in continuous networks; however, it has reasonable drawbacks with discrete networks, while the tree–seed and whale optimization algorithms fit such conditions. On the other hand, these three methods do not show a fluctuating performance compared to one another based on the locating transportation facilities, and thus they deliver similar performance. Besides, although the value of accuracy is high with the application of the conventional technique Weiszfeld algorithm, it does not provide a significant performance accuracy advantage over the meta-heuristic methods.

## Introduction

The meta-heuristics for engineering design problems are significantly powerful tools for detecting optima and have been effectively used for obtaining engineering solutions. Although optimization is used in many engineering fields, it has been used considerably less in the area that forms the motivation of this study. The motivation for analyzing the problem in this study arises from the requirement of one main facility that comprises several emergency units ready for providing services to any part of the country, rather than making significant investment in each city. Emergency cases may include the times of pandemic diseases, such as COVID-19 (coronavirus); floods; earthquakes; and country-wide fires, such as the series of wildfires in Greece in 2018 and the 2019–20 bushfire season in Australia. The importance of transporting supplies during and after emergency cases associated with a wide-scale disaster is emphasized [1]. Therefore, we focus on determining the most convenient transportation facility location with emergency equipment and staff (TFLEE) for an intervention during or after an emergency case. Additionally, the optimum TFLEE can be achieved on the basis of the importance factors of demand points; notably, the importance factors are related to the spatial parameters of demand points. Accordingly, we assign weights to demand points; these weights are associated with many parameters rather than a single parameter. Because weights are adjusted according to the importance factors provided to them, the spatial position of the most appropriate TFLEE also shifts. This shift is also demonstrated in this study. Moreover, the optimization methodology must be tested using other up-to-date optimization methods, to understand the difference in the significance levels between their findings. For constructing a solution methodology for the problem, based on this motivation, we consider establishing a TFLEE; therefore, a single point that is related to the source points (i.e., demand points) in a network needs to be assigned. To form a solution, several techniques in the literature can be applied. Although the previously proposed applications can be used, they must be examined using state-of-the-art optimization techniques.

Meta-heuristic algorithms are effective methods for solving many challenging optimization problems, especially in engineering. For example, the tree–seed algorithm (TSA) is one of the current meta-heuristic methods and has been successful in optimizing different design problems, including transportation. Therefore, TSA was used to solve the present problem. However, since TSA contains stochastic operations like other meta-heuristic methods, performance fluctuations can be observed in different problems. For this reason, to better evaluate TSA’s performance in the current problem, the whale optimization algorithm (WOA), which has performed well in solving complex problems, and Weiszfeld algorithm (WA), which is used in solving transportation-based optimization problems, were preferred in this study.

A renowned technique considers the weights of demand points. It minimizes the Euclidean distances from the weighted demand points to a single location in a two-dimensional (2D) space. It was first developed by Alfred Weber in 1909, to be subsequently called the Weber problem (WP) [2–5]. Because the weights of the source points (i.e., demand points) in a network affect the optimal location of the sink point (i.e., the final location of the facility), an iterative technique can be applied to obtain the optimal location fit for the best service in the network. One of the most accurate and reliable iterative mathematical algorithms for evaluating the optimal facility location is the Weiszfeld algorithm (WA) [6–8]. By implementing WA for solving WP, the transportation information of a network can be analyzed to find the optimal TFLEE. WA has been widely applied to solve WP. Importantly, this application can be tested using recently proposed optimization algorithms, such as the tree–seed algorithm (TSA) [9], and the whale optimization algorithm (WOA) [10]. Methodologies such as WA is able to find two types of data such as *X* coordinate and *Y* coordinate. WA is really fast to find optimal locations; however, WA has essential difficulties to process data in discrete problems. It can find viable solutions with relatively small size data, but big data are seriously challenging for it. As the data size increases, effectiveness of WA decreases. This shows WA is not a flexible methodology. However, TSA and WOA are able to adapt to handle big data and large discrete problems. Flexibility of TSA and WOA is conspicuously an advantage against the number of parameters increase. Because of all of these characteristics, we need to directly compare WA, TSA, and WOA. If we do not, we would not be able to realize the importance of extending the number of parameters for a better analysis. Thus, this comparison can let us reveal true spatial positions of TFLEEs successfully.

Optimization is used not only for determining optimal locations but also for finding solutions in various engineering fields. The optimization of properties, as well as costs, of manufacturing products with application of methodologies is commonly used [11–14]. In spatial studies, performing optimization by using powerful methodologies has recently become common. For instance, a mathematical model to spatially optimize the positions of crew response in an oil-spill emergency case was developed [15]. Moreover, online facility location problem was inspected and the location of a new facility in metric space was determined [16]. Particularly, optimization and meta-heuristics have been commonly used in the area of transportation for decades. Off late, for instance, optimization techniques to acknowledge the most important combination of sensitive connections in a transportation-network topology were used [17]. A methodology that optimized the arrangement of transportation-activity categories was established by maintaining an optimal balance [18]. Urban transport-optimization problems, including time-dependent decision variables and differentiable constraints, were dealt with and a model for solving dynamic optimization problems was proposed [19]. An optimization model was introduced to evaluate the number of bicycle stations required in a large city [20]. The meetup locations on a road network topology were optimized for multiple moving objects or road users, by using Manhattan and network distances [21]. New retail arrangements were explored between train stations by using pedestrian and traffic flow optimization, and combined theoretical perception and empirical outputs [22]. Recently, in the domain of air transport, an optimization model was developed to analyze the performance of an available air service by considering the spatial characteristics [23]. The optimization of the airplane-boarding process was researched on the basis of customer reactions [24], while a model that minimized differences in flight hours for each aircraft in the upcoming time periods was created by using constraints based on the crew workload [25]. Additionally, the emergency response and evacuation policies were optimized by considering parameters including congestion level, queues, and vehicle removal [26].

In addition, in the field of transportation, some researchers work on typical transport facility location problems. For example, charging facility locations are optimized according to many criteria for electric vehicles [27–29]. Furthermore, they consider several measures such as traffic flow, recharging vehicle battery demand, and network capacity constraints to attract more vehicles to the charging facilities, maximize their revenue, and minimize public social costs. Besides all these, some studies formulate techniques and models to distribute bikes or repairable service parts and their facility locations through networks providing proper allocation strategies and spatial distribution for servicing the maximum area with minimum resources [30–32]. Furthermore, many other techniques are commonly used for location analyses. For instance, new industrial paths related to regional challenges were investigated and the path transformation in a country was explained [33]. For locating a facility in a network, replacing or renewing the locations of various facilities was studied by using several techniques [34–37]. To locate a facility in a transportation network, the central median location problem can be used. To assess a solution using the central median location problem, the sum of the geometric planar distances from the demand points to the facility location can be minimized [38]. Thus, the location of the final facility can be computed. Although this technique seems straightforward and relevant, each demanding network point’s weight is not examined. Besides the central median location problem, other types of problems have also been used in the literature. For example, the single-allocation hub median location problem was analyzed by combining two heuristics–simulated annealing and ant colony optimization [39]. For emergency-service systems, the effect of a backup service through *p*-median location problems was studied by considering different demand policies, and found the best backup service [40]. Additionally, in many studies, the number of parameters, and thus, the importance of parameter weights, were increased to demonstrate the high performance of location-analysis techniques and discover more viable results [41, 42].

WP is an operation research problem for optimizing the location of a new node in a network. Its principal purpose is to minimize the Euclidean distances from a network’s demand nodes, which have specific weights, to a new service point, which is actually an optimal location [2, 3, 43]. A popular and powerful iterative method of WA was applied to solve WP with confidence [7, 8, 44, 45]. In recent years, many studies based on WP have especially addressed WA to solve WP. For instance, WP was generalized using box constraints, and a projected WA was applied with a fixed point for iterations [46]. The solution style of WP was improved by introducing a parabolic approximation of the objective function, and an effective way of solving WP was developed by using a WA structure fortified with few demand points [47]. A geometric interpretation of WA convergence was introduced to answer the final position of a point in a problem constructed by WP [6]. However, the results of the standard Newton’s method on WP were compared with the ones calculated using WA [48]. Conclusion was Newton’s method was significantly more efficient than WA. Using WA, an appropriate location for a cement plant was computed according to the locations of manufacturing goods [49]. A new methodology was proposed to solve WP and the inadequate ways of WA were emphasized while the great-circle distance was used as an input measure [50]. A precise solution algorithm for WP was developed by using the tools of a geographic information system, and the convenience of the approach was demonstrated through a practical application [51].

TSA is an iterative methodology like WA, inspired by the spreading of tree seeds in the nature and their germination levels under appropriate conditions [9]. Various studies have been conducted using TSA for solving many engineering problems. Models were created as control schemes for optimization techniques with particular parameters [52–56]. Binary optimization problems were solved using TSA, and it was shown that the hybrid TSA type provided satisfactory quality of results [57, 58]. A method that incorporated ordinal optimization into TSA was proposed to solve simulation optimization problems subject to chance variation [59]. It was compared with heuristic methods and satisfactory computing efficiency was achieved. TSA was operated for tuning a function network for segmentation [60], while the problem of optimal electric power flow in energy network systems was solved by applying TSA [54]. Structural-damage-identification problems were inspected by applying a TSA that was improved using the Gaussian bare-bone mechanism/algorithm and withering [61]. TSA was used to solve optimization problems, including a nonlinear hysteretic parameter identification problem, and improvements were observed in the accuracy of the findings compared to the previous studies [62, 63]. Then a discrete version of TSA was proposed to calculate the optimized solutions of permutation-coded problems [64].

WOA is based on nature and inspired by humpback whales’ bubble-net activity [10]. The performance of this algorithm for transport facility location analysis is a matter of priority because it offers advanced features for solving such situations. However, the minimal study included WOA application to find solutions to real-life transport facility location problems, although many employed the WOA technique to solve a significant number of engineering problems [65–74].

The novelty of this study arises from addressing the following concepts. Firstly, it is the first study comparing WA, TSA, and WOA performances in the area of transportation. Secondly, a novel way of revealing the insight of testing the importance parameters affecting the determination of TFLEE is offered. Many studies mention the power of meta-heuristic algorithms of TSA and WOA techniques together in their works [75–77]. However, a comparison of WA, TSA, and WOA methods via locating transportation facilities with weightings is made for the first time, thanks to this study. Additionally, one of the most important concerns we wonder about in this research is how close the meta-heuristic methods such as TSA and WOA are to WA, which is successful for the transportation problems [49, 78–80]. In accordance with this purpose, although this article does not offer a development of any modification or improvement of the existing algorithms, this paper provides an important insight into the accuracy of the meta-heuristic methods against the typical transportation location problems solving algorithm of WA.

This paper is organized as follows. The Materials and Methods section explains the optimization problem structure and the optimization methods to be applied. Then, while the dataset and application details are introduced in the Application Instance section, the results and related comments are discussed in the Results and Discussion part. Subsequently, the conclusions are given with future work. After the reference list, a supporting table including detailed outputs is provided.

## Materials and methods

The optimization problem of this study can be introduced as follows, as proposed in the study of Demir in 2021 [81]. However, the main difference between this study’s problem and the problem of Demir [81] is as follows: the comparisons between different optimization techniques take place in this research, while only one technique is considered for the evaluation in the study of Demir [81]. The problem seeks the most suitable (*X*, *Y*) coordinates in such a way as to minimize the value of *d*(*X*, *Y*) and satisfy the condition of *X* < *x*_*n*_, *X* > *x*_*s*_, *Y* < *y*_*e*_, and *Y* > *y*_*w*_. The problem can be introduced in **Eq 1**.


$$Minimize\;d\left(X,Y\right)=\overset{n_{a}}{\underset{i=1}{\sum }}W_{i}\sqrt{{\left(X-x_{i}\right)}^{2}+{\left(Y-y_{i}\right)}^{2}}$$ (1)

where;

*d*: distance value

*i*: airport index

*n*_*a*_: the number of airports

*x*_*i*_, *y*_*i*_: *x* and *y* coordinates of airport *i*, respectively

*X*, *Y*: *x* and *y* coordinates of candidate location, respectively

*W*_*i*_: the weight for airport *i*

*x*_*n*_, *x*_*s*_: the northernmost and southernmost airport, respectively

*y*_*e*_, *y*_*w*_: the easternmost airport and the westernmost airport, respectively

### Optimization methods

#### Weiszfeld algorithm

To solve WPs, let us suppose *n* demand points in a network of *L*_*i*_ = (*x*_*i*_, *y*_*i*_), where *i* = 1, …, *n*, on the surface of ℝ^*δ*^, assuming *n* ≥ 3 and *δ* = 2. Accordingly, let all demand points have the positive weights of *a*_*i*_ engaged with a specific demand point of *i*, where *a*_*i*_ ≥ 0. If the position of the new facility is *L* = (*x*, *y*) and if the Euclidean distance (i.e., cost) between the facility location and demand point *i* is *c*_*i*_(*L*), the following cost function *f*(*L*) can be minimized, and thus, WP can be solved. Consequently, the minimization of *f*(*L*) in **Eq 2** ascertains the most appropriate place of *L* = (*x*, *y*) [2–8].


$$f\left(L\right)=\overset{n}{\underset{i=1}{\sum }}a_{i}\left[c_{i}\left(L\right)\right]$$ (2)

The location-allocation problem is handled by WA, which iterates the optimization technique for determining a stationary point of a given objective function. Fundamentally, once WA is applied, it minimizes the following **Eq 3**, and thus, the optimal location can be detected [2–8].

$$f\left(S\right)=\overset{m}{\underset{j=1}{\sum }}\omega_{i}C_{i}$$ (3)

where *f*(*S*) denotes the final site location and *ω*_*i*_ *C*_*i*_ denotes the product of the specific weight of demand point *j* (e.g., customer point *j*) and the cost of distance (i.e., the Euclidean distance cost) of traveling from demand point *j* to the final site location (*X*_*f*_, *Y*_*f*_) on ℝ^2^. The term *m* denotes the number of points or customers that demand/request a service. Moreover, let the final site location satisfy the inequality (*X*_*f*_, *Y*_*f*_) ≥ 0, where the final output must lie on a fixed network topology. Besides this typical methodology, we introduce the weights of demand points. The weights are associated with a couple of parameters related to the demand points, rather than considering a single parameter for deciding the weight of a demand point. Conversely, the optimum location of the facility can be attained on the basis of importance factors of the demand points. The importance factors are relevant to the spatial parameters of the demand points. Accordingly, let *α*_*jk*_ be a positive weight of demand point *j* according to parameter *k*, and let $\omega_{j}=\sum_{k=1}^{l}\alpha_{jk}$, where ∀*j* = 1,2, …, *m* and *ω*_*j*_ ≥ 0, where *ω*_*j*_ ∈ ℚ. Therefore, the weights are diversified using several parameters associated with the demand points. The methodology of solving WP using the iterative technique of WA can then be implemented. The iteration continues until the same output or a similar result is consistently produced. Thereafter, the iterations for the optimization finish, and the final outcome is reported.

#### Tree–seed algorithm

TSA, which is a meta-heuristic technique, is considered in this study to appraise the results of WP that is solved using WA. The main reason for this is the easy adaptation of the problem in this study to TSA. For example, candidate location and solution position in the problem denote candidate solution and seed position in TSA, respectively. Meanwhile, coordinates of candidate locations represent coordinates of seed positions which are design variables. Objective values (*OVs*) in the problem solution indicate performance of TSA.

As seen in **Table 1**, the problem in **Eq 1**, in which our aim is explained, can be adapted to the working principle of TSA [9]. Although the candidate airport in our problem is also the candidate solution of the TSA method, the position of the tree–seed in TSA reflects the airport location (**Table 1**). Moreover, the candidate airport location coordinates are the coordinates of the tree–seed locations, which are the design variables. The performance of TSA is to find the value of *d*, which is the aim of our problem.

**Table 1:** Adapting the study to the TSA.

In our problem	Represented in TSA
Candidate airport	Candidate solution in TSA
Airport location	Tree–seed location
Candidate airport location coordinates (*X*, *Y*)	Tree–seed location coordinates (design variables)
*d* value	Performance of TSA (objective)

**Fig 1:**
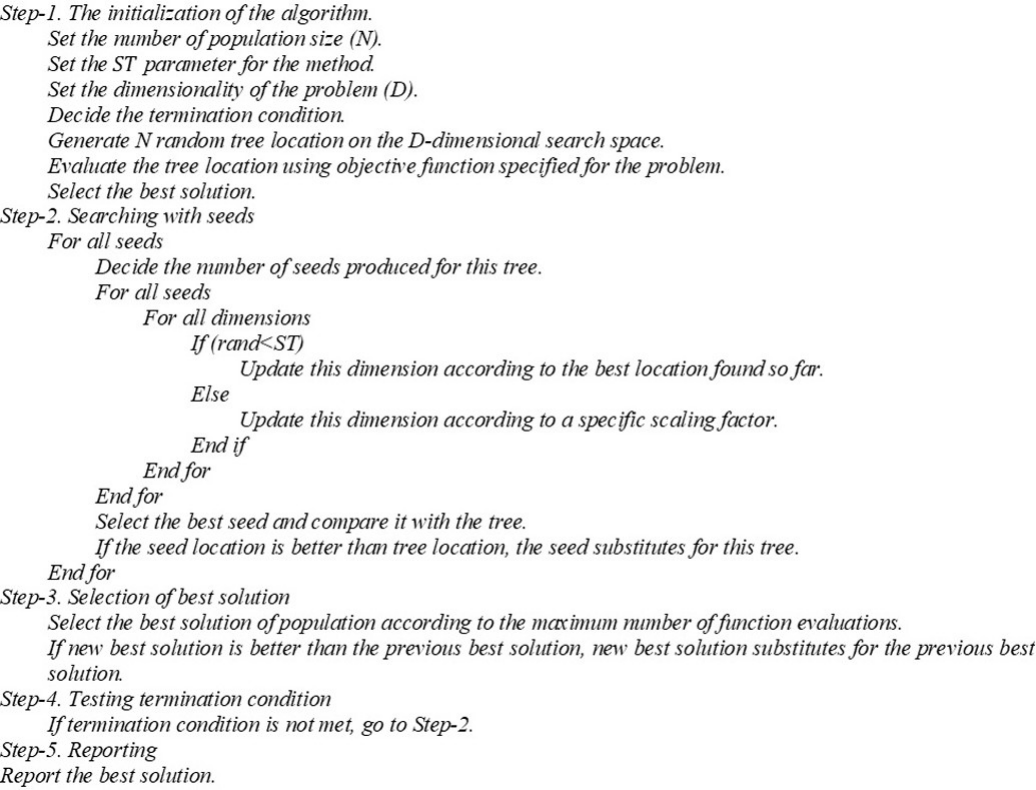
The pseudocode of TSA [9].

The working principle of TSA is as follows [9]. In nature, trees grow and multiply in a region by spreading their seeds. Via stochastic results, the seeds transfer to the regions that are far from their mother trees. The casual agents of such seed transfers may be sometimes wind or even animals that move or fly around. Thus, seeds can travel miles away from their mother trees. However, not all of them can germinate and start to grow. Only the ones that receive ideal conditions can germinate. TSA was inspired from such seed-based occurrences. In TSA, a tree is defined as solution vector, while a seed is called a candidate solution, and the location phrase is described as design variables. Additionally, the performance of TSA is evaluated on the basis of its capability of search tendency *ST* that is to regulate the stochastic spread rate of the seeds. Here, the main steps involved in TSA for this study are presented, as indicated in the work of Kiran in 2015 in **Fig 1** [9].

Initialization of the algorithm is step-1, which outlines the search and problem parameters. *N*_*pop*_ denotes the number of trees, and *s*_*min*_ and *s*_*max*_ denote the minimum and maximum numbers of seeds on a tree, respectively. Additionally, *Iter*_*max*_ denotes the maximum number of iterations. Subsequently, through the application of **Eq 4** satisfied by **Eqs 5** and **6**, the initial solutions are haphazardly produced [9]. Subsequently, the algorithm memory stores the findings of evaluations and the fitness values of the results.


$$X_{i,j}=round\left(1+N_{sta}\cdot rnd\left(0,1\right)\right)$$ (4)

$$i=1,2,\ldots ,N_{pop}$$ (5)

$$j=1,2,\ldots ,N_{sta}$$ (6)

In **Eq 4**, *X* denotes the matrix in the algorithm memory, which contains design variable values provided by the solutions. Additionally, *rnd*(0,1) denotes a mathematical function that generates a random real number between 0 and 1. Additionally, *round* signifies another function that rounds the real value assigned to the nearest integer value in **Eq 5**. *N*_*sta*_ in **Eq 6** indicates the number of stations in the network considered for the optimal solution.

Step-2 is the search using seeds, where seeds are the candidate solutions for the problem. Seeds are developed on each tree as a pool of solutions. The number of seeds on a tree, *N*_*seed*_, is between *s*_*min*_ and *s*_*max*_. Accordingly, the values assigned to the seeds are computed as follows [9]:

$$S_{k,j}=X_{i,j}+rnd\left(-1,1\right)\cdot \left(X_{best,j}-X_{r,j}\right)$$ (7)

$$S_{i,j}=X_{i,j}+rnd\left(-1,1\right)\cdot \left(X_{i,j}-X_{r,j}\right)$$ (8)

$$i,r\in 1,2,\ldots ,N_{pop}\;,\;i\neq r$$ (9)

$$k=1,2,\ldots ,N_{seed}$$ (10)

$$j=1,2,\ldots ,N_{sta}$$ (11)

If *rnd* < *ST*, then **Eq 7** applies. However, if *rnd* ≥ *ST*, then **Eq 8** applies. Notably, **Eqs 9–11** apply for **Eqs 7** and 8. The term *i* denotes the index of the currently selected tree, *r* indicates the index of a randomly chosen tree, and *S* indicates the seed matrix of the *i*th tree. Accordingly, *X*_*best*_ denotes the best solution found in the solution pool [9].

In step-3, the seeds produced by the trees are evaluated for fitness for plantation/germination. Accordingly, the best seeds replace their mother trees when they find the ideal conditions. The evaluations in Steps-2 and -3 are processed via TSA until the total number of evaluations becomes *Iter*_*max*_.

#### Whale optimization algorithm

**Fig 2:**
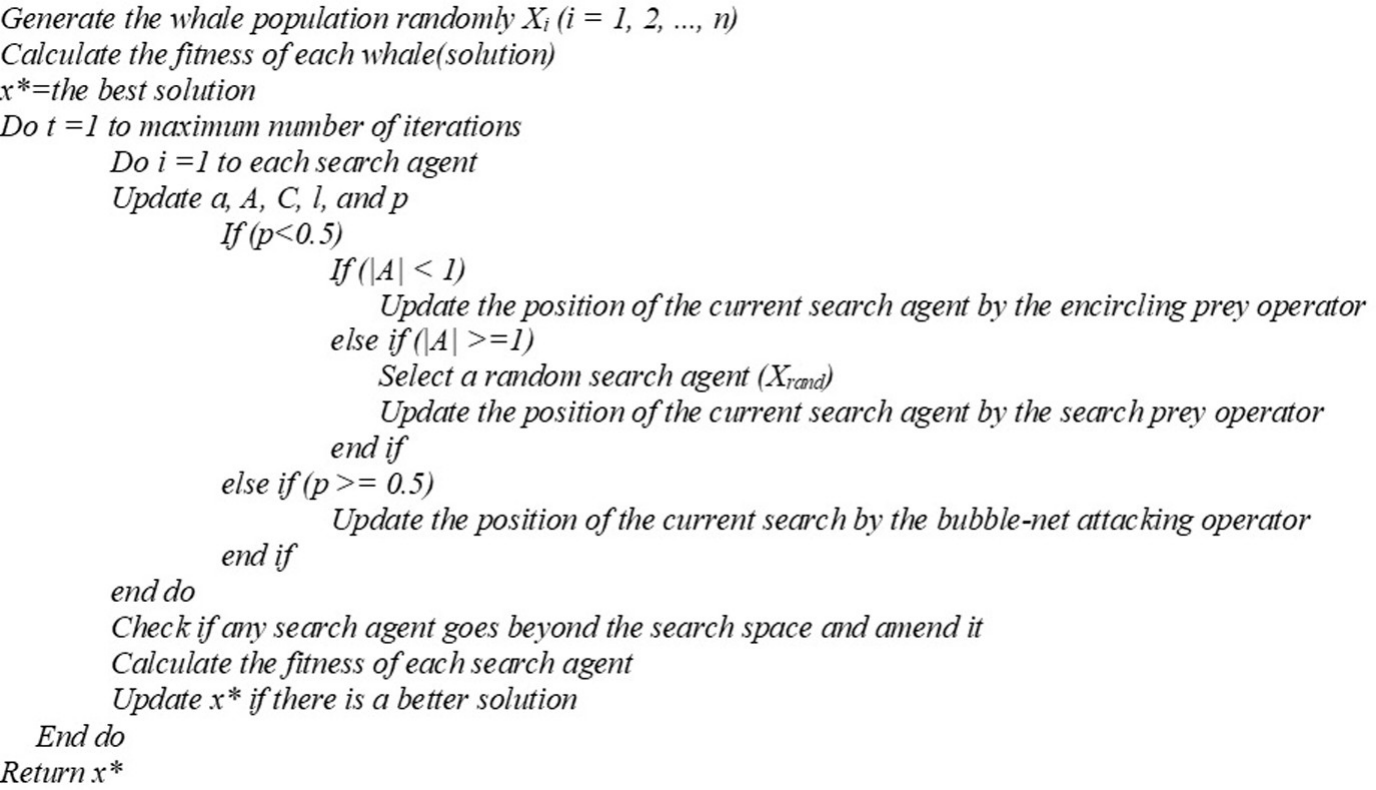
The pseudocode of WOA [10].

Mirjalili and Lewis introduced a singular objective algorithm that is a nature-inspired and stochastic population-based technique WOA [10]. The algorithm was inspired by humpback whales’ spiral bubble-net predation approach to grabbing their prey. WOA is one of the powerful and fresh-built meta-heuristic techniques, whose pseudocode is presented in **Fig 2**. Furthermore, the effectiveness of WOA in engineering problems such as optimizing complex frameworks has been demonstrated. Accordingly, the robustness of WOA has already been tested in a recent study [82], and the algorithm details are as follows.

Step-1 is ‘Generating the initial solutions’. The initial solutions in the WOA memory are generated, calculated, and stored based on a process evaluating the opening solutions and computing their fitness values [10]. Also, the unfeasible solutions and their fitness values are kept in the algorithm memory.

Step-2 is ‘Updating the solution’. WOA determines the kind of operator to apply [10]. For this case, either ‘encircling prey’ or ‘search prey’ are chosen, or the ‘bubble-net attacking’ operator is used (**Eq 12**). The parameter H in **Eq 12** is computed in **Eq 13**, where *h* is linearly decreased starting from 2.0 to the value of 0.0 during the iterations. This reduction happens not only in the exploration phase but also in the exploitation stage.


$$\begin{array}{l} If\;rand\left(0,1\right)<0.5\\ \;if\;|H<1|\;\to \;use\;the\;encircling\;prey\;operator\\ \;if\;|H\geq 1|\;\to \;use\;the\;search\;prey\;operator\\ else\\ Use\;the\;bubble-net\;attacking\;operator \end{array}$$ (12)

$$Η=2h\;rand\left(0,1\right)+h$$ (13)

The solution is updated in the encircling prey period (**Eq 14**), where $v_{j}^{best}$ is the *j*th design variable of the optimum solution in the WOA memory, and *n*_*dv*_ is the number of design variables. Also, *K* is the control parameter computed in **Eq 15** [10].


$$v_{j}^{new}=v_{j}-Η\;\left|K\;v_{j}^{best}-v_{j}\right|;j=1,2,\ldots ,n_{dv}$$ (14)

$$K=2\;rand\left(0,1\right)$$ (15)

On the other hand, WOA shifts the solution according to randomly chosen solutions in the search prey section (**Eq 16**), where $v_{j}^{rand}$ is the *j*th design variable of the random solution in the memory [10].


$$v_{j}^{new}=v_{j}-Η\;\left|K\;v_{j}^{rand}-v_{j}\right|;\;j=1,2,\ldots ,n_{dv}$$ (16)

If the previous two operators are not applied, the bubble-attacking phase takes place, where the spiral formula is utilized to amend the solution (**Eq 17**) [10]. In this phase, where *β* is the constant or invariable factor fixed at 1.0 to specify the logarithmic spiral shape, *D* is the distance vector for the distance between the whale and prey, and *l* is a random value between an interval of [–1, 1].


$$v_{j}^{new}=e^{\beta l}cos\left(2\pi l\right)D_{j}+v_{j};j=1,2,\ldots ,n_{dv};\;l=\left(2rand\left(0,1\right)-1\right)$$ (17)

Step-3 is ‘Refreshing the memory’. The new solutions’ fitness values are compared to those of prior versions. Then, they replace old versions with new ones if the new solutions own better fitness values. After that, WOA returns to step 2, and all operations between steps 2 and 3 continue until the maximum number of iterations is achieved. At this point, an iteration is defined as the completion of one solution assessment [10].

In addition, the design in our problem is represented in WOA as the whale (**Table 2**). While the candidate airport location coordinates are the whale location coordinates, which are the design variables, the performance of WOA is to determine the objective value of *d* in our problem.

**Table 2:** Adapting the study to the WOA.

In our problem	Represented in WOA
Design	Whale in WOA
Candidate airport location coordinates (*X*, *Y*)	Whale location coordinates (design variables)
*d* value	Performance of the whale (objective)

Determining the parameters and changing them according to the problem’s structure is essential in such stochastic algorithms. Therefore, carrying out a sensitivity analysis is worthwhile in this study. However, our study did not perform this analysis because WA, TSA, and WOA techniques were applied to many other engineering problems. For instance, based on the reference studies [65, 74, 82, 83], the parameters of TSA and WOA were utilized to assess the network problem solutions. Moreover, since the parameters in WA were previously used in the analyses [47, 80, 81], they were employed in our work based on the merit in the aforementioned studies. Accordingly, in the following, the parameters involved in the outputs are provided (**Table 3**).

**Table 3:** Parameters involved in the results.

Optimization algorithm	Search parameters
TSA	Population size = 30
	Search tendency = 0.5
	Low number of seeds produced by a tree = 3
	High number of seeds produced by a tree = 8
WOA	Population size = 30

**Fig 3:**
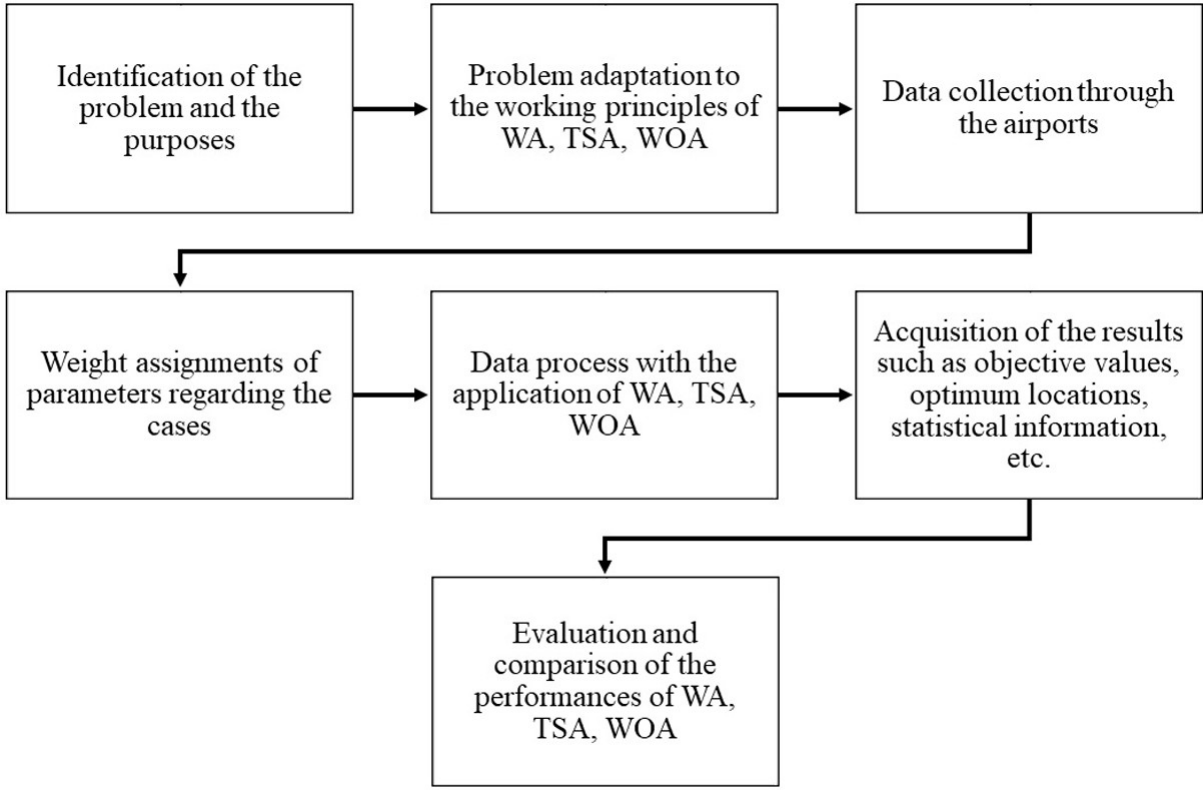
Present work procedure flowchart.

In this study, the present work procedure is exhibited using a flowchart (**Fig 3**) to identify crucial design steps and provide a larger picture of the process.

## Application instance

The diversity of the weights has become an important input for many transportation analyses and resolving optimization problems. However, the number of different weights is underestimated, particularly in the analyses of aviation-transportation locations. In this section, an empirical application for understanding the importance of the weights of demand locations. The operability of the proposed methodology for determining a new TFLEE is presented. The scope of this study includes various parameters not only related to local properties but also work together. Topological properties of transportation networks are very important [84, 85]. The parameters are briefly introduced as follows. The first parameter is the number of passengers that stop by the airport in 12 months, called parameter-*1* (*P1*) in this article. The second parameter is the airport size, called parameter-*2* (*P2*). The third parameter is the number of days with precipitation, such as rainfall and snowfall. It is called parameter-*3* (*P3*), which denotes unpleasant weather conditions for both landing and take-off. All three parameters demonstrating topological properties decide the attractiveness of an airport and have special weights to give priority to an airport while making decisive outputs.

For dataset *P1*, we use the number of airline passengers because the more the number of passengers at an airport, the more likely are the casualties (e.g., infection in the case of pandemics, such as COVID-19) to occur at that particular airport in case of an event. The vulnerability of airports and high risk of casualties in the case of an attack were proved [86]. Therefore, emergency services must be maximized at busy or populated airports. The time period of the data is from October 2018 to September 2019, i.e., 12 months. In total, 55 civil airports in Turkey are focused to gather the data. **Table 4**, as a sample of dataset *P1*, presents the total number of passengers that visited six airports in October 2018. Accordingly, it presents a sample dataset for the approximate areas of six airports, and the average number of precipitation days of six cities to which the airports belong.

**Table 4:** Data sample for the total number of airline passengers at six airports (general directorate of state airports authority, 2019), approximate-area measurements, and average number of precipitation days of six airports (Turkish State meteorological service, 2019).

	Airport Code					
	IST	SAW	ESB	ADB	AYT	DLM
Number of passengers in October 2018	57,737,038	28,882,781	14,407,609	11,570,525	29,502,441	4,409,521
Approximate area (km^2^)	25.022	8.561	7.530	6.782	13.229	5.760
Number of days with precipitation	128.4	128.4	103.2	78.0	74.0	93.4

Monthly passenger counts are considered and 12-month data are summed up to obtain an annual passenger count for a particular airport. The values for the number of passengers are then normalized according to the highest number of passengers, among all airports. The highest value is set to 100 and the remaining values are assigned a value out of 100, according to their real magnitudes. Thus, the values of *P1* are weighted as a parameter of attraction for further analysis.

The dataset *P2* shows sizes of the area/land under airport use. The terrain area size of an air-field parameter must be included in the analysis because wider airports are generally more likely to host more passengers because of busy traffic. Thus, more casualties or deaths can be observed in major airports or regions around them [87]. To gather dataset *P2*, the areas of all 55 airports are measured using an online area calculator [88]. Subsequently, the area values are normalized as happened for *P1*. To indicate the attractiveness of an airport according to *P2*, such example can be provided. According to **Table 4**, airport SAW is more attractive than airport DLM, as the area of the former is greater than that of the latter. Likewise, airport ADB is less attractive than, for instance, airport AYT in terms of the airport-land-use area.

In dataset *P3*, the average number of precipitation days in a year observed at these airports is important. This is because the worse is the weather condition of an airport, the higher is the risk for an accident [89, 90]. Pleasant weather conditions are preferable over bad one for safe landings and take-offs. Therefore, the attractiveness or importance values of the airports that have bad weather conditions all year-round increase. The data for the average number of precipitation days are provided by an official meteorological service. Subsequently, the average number of days with precipitation is normalized according to their highest value. Thus, the weighted values of *P3* are all set in terms of their attractiveness. For example, airport ESB is more attractive than airport DLM in terms of weather conditions, as the average number of precipitation days for the former is more than that of the latter (**Table 4**).

### Data assignments

There exist, for this study, three spatial parameters that affect the weight of the attractiveness of each airport. Each parameter can be more important than the other. Therefore, we must assign weights to the parameters to determine the final weight of a particular airport. To that end, we prioritize the parameters using percentages. Accordingly, each parameter is assigned some percentage weight, and the analysis can provide us results that indicate the optimal spatial locations for each case.

**Fig 4:**
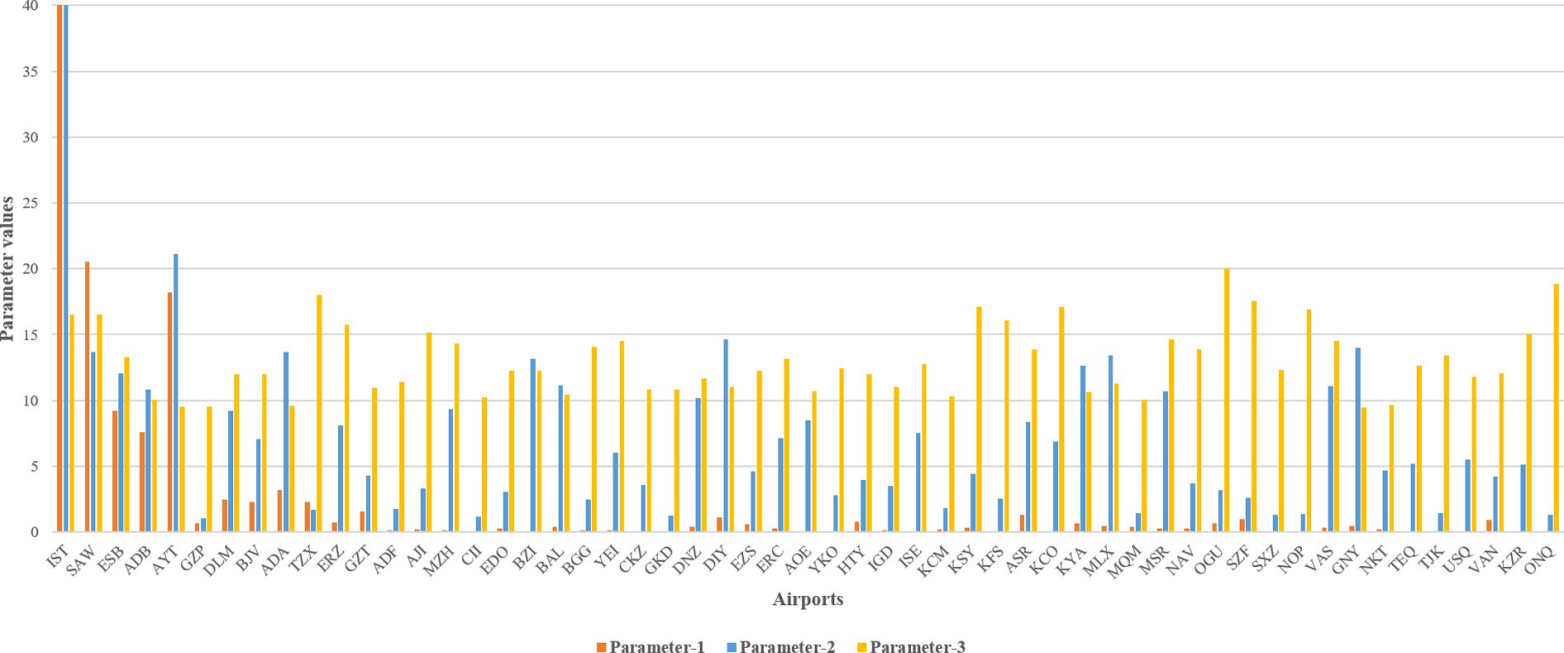
For Case-*24*, the values of three parameters that correspond to the airports.

Because there exist three parameters, their total percentage weights must always be 100%. For instance, if the weight of *P1* is 80% and that of *P2* is 10%, then the weight of *P3* must be 10%. Therefore, if the cases are set using this percentage distribution, there will be 66 cases that represent the weight assignment of the parameters. All of the weight assignments of parameters regarding the cases are depicted in **S1 Table**. The increment value of the percentage assignments will be 10%. Clearly, the increment value (i.e., presently 10%) may be smaller, such as 5%, or larger, such as 20%; however, the increment value is not the focus of this study. **Table 5** presents a couple of sample cases that demonstrate the weight assignment of parameters. According to **Table 5**, the 66 cases correspond to 66 different scenarios depending on the importance weight. For instance, **Fig 4** depicts the values of the three parameters for an example scenario of Case-*24* concerning the airports according to the importance percentages in **Tables 5** and **S1**.

**Table 5:** Cases established using percentage assignments.

Cases	% of *P1*	% of *P2*	% of *P3*	Optimum location
Case-*1*	100	0	0	*L*_1_
Case-*2*	90	10	0	*L*_2_
Case-*3*	90	0	10	*L*_3_
Case-*4*	80	20	0	*L*_4_
Case-*5*	80	10	10	*L*_5_
Case-*6*	80	0	20	*L*_6_
Case-*7*	70	30	0	*L*_7_
Case-*8*	70	20	10	*L*_8_
⁞	⁞	⁞	⁞	⁞
Case-*24*	40	40	20	*L*_24_
⁞	⁞	⁞	⁞	⁞
Case-*64*	0	20	80	*L*_64_
Case-*65*	0	10	90	*L*_65_
Case-*66*	0	0	100	*L*_66_

As can be seen from **Fig 4**, while the values of *P1* are significantly diverse, those of *P2* and *P3* diversify less, especially the ones corresponding to *P3*. Moreover, *P1* and *P2* are not closely related, although both have the same importance, i.e., 40% each. This might be because the passenger crowd at an airport does not indicate the magnitude of the physical area of that airport, and vice versa.

Let us inspect the airport IST under Case-*24* in **Fig 4**. The importance distribution in Case-*24* is 40% for *P1*, 40% for *P2*, and 20% for *P3* (**Table 5**). Therefore, the airport IST has importance values of 40 points out of 40 points for *P1* (because it has the highest value for the number of passengers), 40 points out of 40 points for *P2* (because it has the largest size of the area under airport use), and 16.504 points out of 20 points for *P3* (because it has a high value for the average number of precipitation days in a year) (**Fig 4**). Therefore, the total weight of airport IST in Case-*24* is 96.504 points out of 100 points, given that the highest possible point that an airport can get in a particular case is 100 points. Moreover, inspecting the airport AYT in **Fig 4** gives another pattern. For example, the airport AYT gets the following importance values in Case-*24* (**Fig 4**). 18.198 points out of 40 points for *P1* (because it is the third busiest airport based on the number of passengers), 21.148 points out of 40 points for *P2* (which is the second-highest value for *P2* in Case-*24* because it has the second largest size of the area under airport use), and 9.512 points out of 20 points for *P3* (because it has a moderate value for the average number of precipitation days in a year). Therefore, the total weight of airport AYT in Case-*24* is 48.858 points out of 100 points. Overall, because there are 66 cases in this study, the charts for other cases look different than in **Fig 4**. Thus, various cases might demonstrate a challenging comparison between WA, TSA, and WOA in this study.

## Results and discussion

The optimal location results for each case obtained from WA, TSA, and WOA are achieved for the 66 cases (i.e., options for siting). Furthermore, all results are recorded and verified. The output records, for example, the digital documents of numeric data, evidently state that the local optimal solutions are discovered with the infeasibility of 0.00 for every case. Although the output reports for *L*_1_, *L*_2_, …, *L*_66_ denote that each solution is a local minimum, that local minimum is actually the global minimum because the problem is convex. Consequently, in this study, the results are verified to be the optimal solutions using the WA, TSA, and WOA methods.

**Fig 5:**
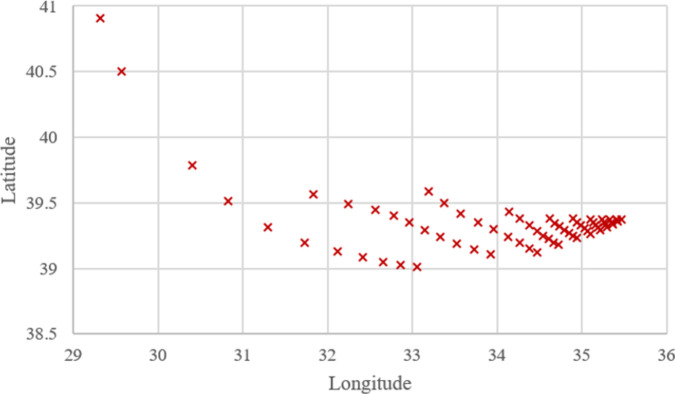
Optimum locations computed for 66 cases using WA.

As seen from **Fig 5**, the effect of *P1* is the highest at the beginning (i.e., 100% for Case-*1*) because the other parameters have no percentage, meaning their attractiveness or importance values are zero. Therefore, the optimum location *L*_1_ is significantly close to the busiest airport of the country, IST. The positions of the optimal locations generally move toward the south-east of the country as the attractiveness values of parameters *P2* and *P3* increase. One of the reasons for this situation is the increase in dryness in the weather with traveling south. Another reason is that the physical area sizes of most airports in the country are almost equal; therefore, their *P2* attractiveness values are close to each other. An example shows that location *L*_50_ is in the east of location *L*_4_ because more importance is given to *P2* and *P3* in Case-*50* than in Case-*4*. Thus, *L*_4_ can be found in the western part of the country, while *L*_50_ can be located around the middle part of the country.

**Fig 6:**
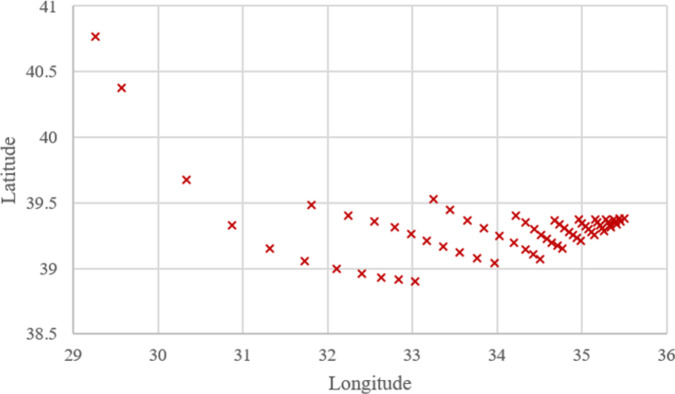
Optimum locations computed for 66 cases using TSA.

**Fig 7:**
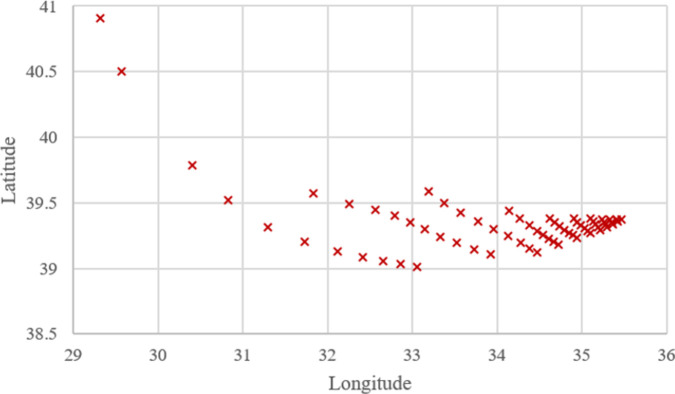
Optimum locations computed for 66 cases using WOA.

**Figs 5–7** show that the locations of the cases resulting from WA, TSA, and WOA are almost similar to each other but not the same. This clearly shows that the *OVs* resulting from WA, TSA, and WOA are not equal. All of the *OV* regarding the cases are presented in **S1 Table**. Additionally, the comparisons between the algorithms in terms of *OV* difference rate is discussed with the help of charts in the upcoming parts of this section.

Moreover, the means of the *OV*s of WA, TSA, and WOA and their standard deviations are computed. Moreover, **Table 6** depicts a comparison between the means of the *OV*of WA, TSA, and WOA. The difference between the means of the result sets is tiny since the mean *OV*s of WA, TSA, and WOA are 170.32, 171.02, and 170.43, respectively. In short, the maximum difference between the means is 0.70. In addition, minimum *OV*_*WA*_ is found as 18.13, while minimum *OV*_*TSA*_ and *OV*_*WOA*_ are discovered as 17.93 and 18.25, respectively. Even so, maximum *OV*_*WA*_ (379.14) is lower than maximum *OV*_*TSA*_ (381.18) and maximum *OV*_*WOA*_ (379.23). The means (170.32, 171.02, and 170.43), the median values (157.98, 158.56, and 158.10), and standard deviations (88.10, 88.67, and 88.09) of *OV*_*WA*_, *OV*_*TSA*_, and *OV*_*WOA*_ respectively are significantly close to each other. However, this raises the question of whether there is a significant difference between the results of the algorithms. Therefore, a *t*-test is applied based on the variable of the application type. The analysis of the independent group of the *t*-test using two samples with equal variance between the *OV*s from two selected algorithms. For instance, the *t* value is computed for the WA and TSA sample couple as 0.96365 (**Table 6**). Additionally, while the *t* value for the TSA and WOA sample couple is calculated as 0.96938, it is found to be 0.99424 for the sample couple of WA and WOA. Therefore, no significant difference between the results of WA and TSA, TSA and WOA, WA and WOA is observed, from the statistical viewpoint.

**Table 6:** Statistical information about the objective values of the algorithms.

	WA	TSA	WOA
Mean *OV*	170.32	171.02	170.43
Standard Deviation	88.10	88.67	88.09
Minimum *OV*	18.13	17.93	18.25
Maximum *OV*	379.14	381.18	379.23
Median *OV*	157.98	158.56	158.10
*t* value (WA-TSA)	0.96365		
*t* value (TSA-WOA)	0.96938		
*t* value (WA-WOA)	0.99424		

**Fig 8:**
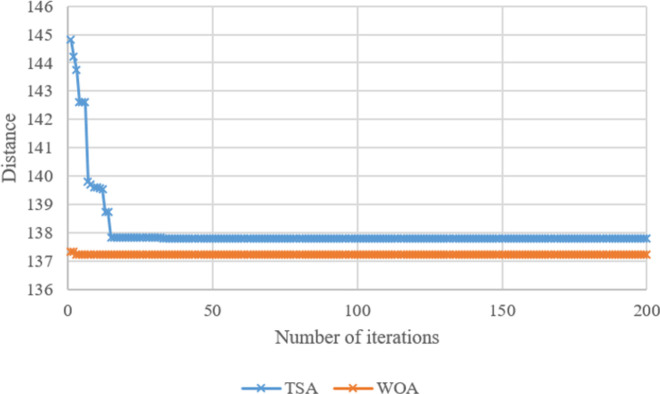
Search history of TSA and WOA for Case-*10*.

**Fig 9:**
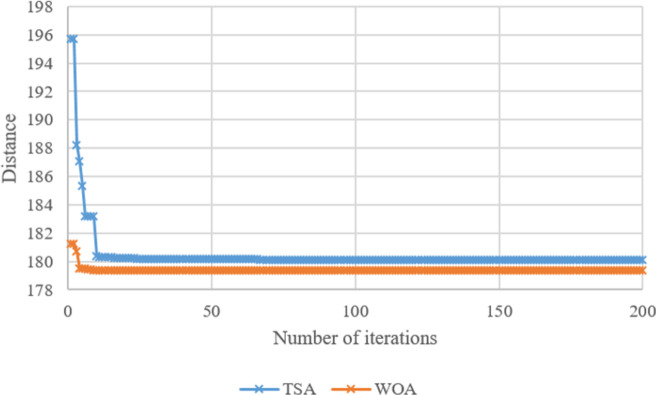
Search history of TSA and WOA for Case-*20*.

**Fig 10:**
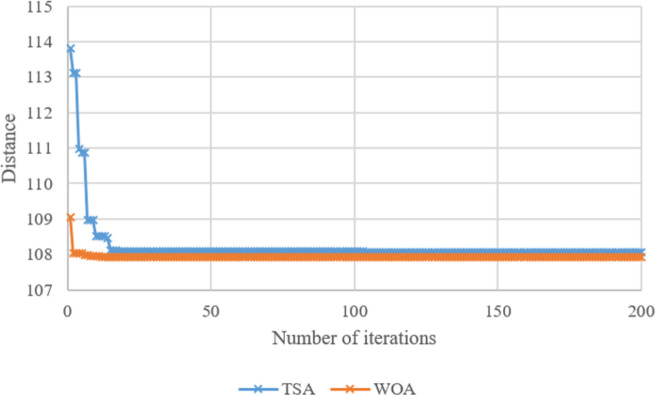
Search history of TSA and WOA for Case-*30*.

**Fig 11:**
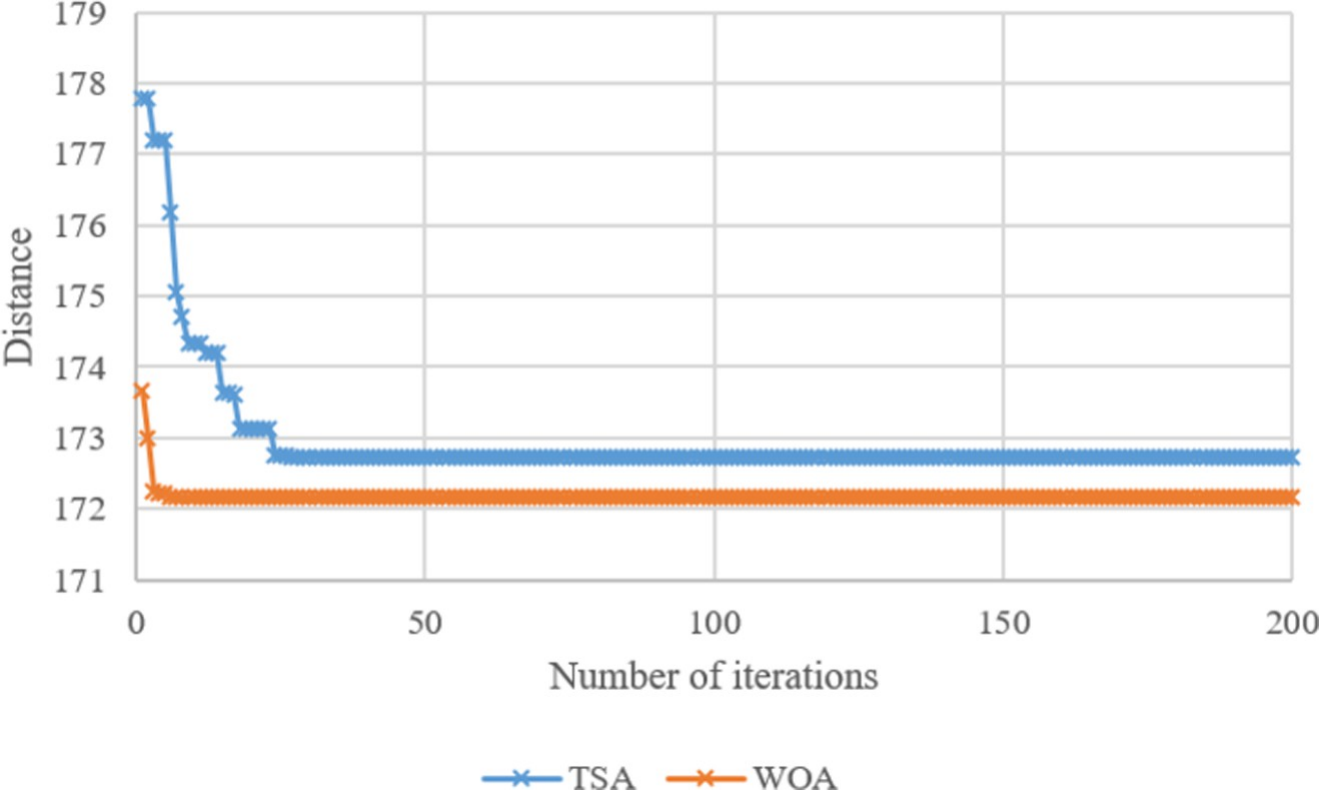
Search history of TSA and WOA for Case-*40*.

**Fig 12:**
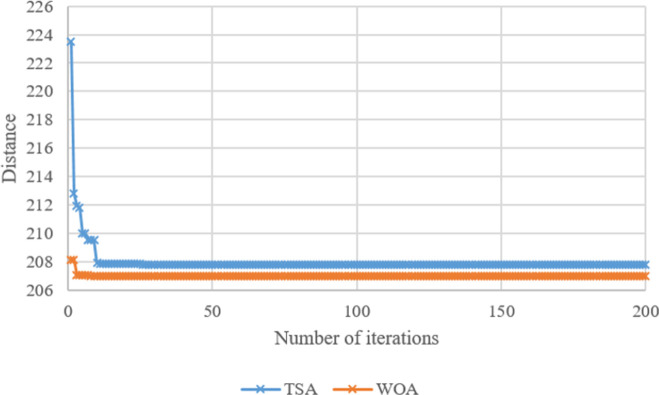
Search history of TSA and WOA for Case-*50*.

**Fig 13:**
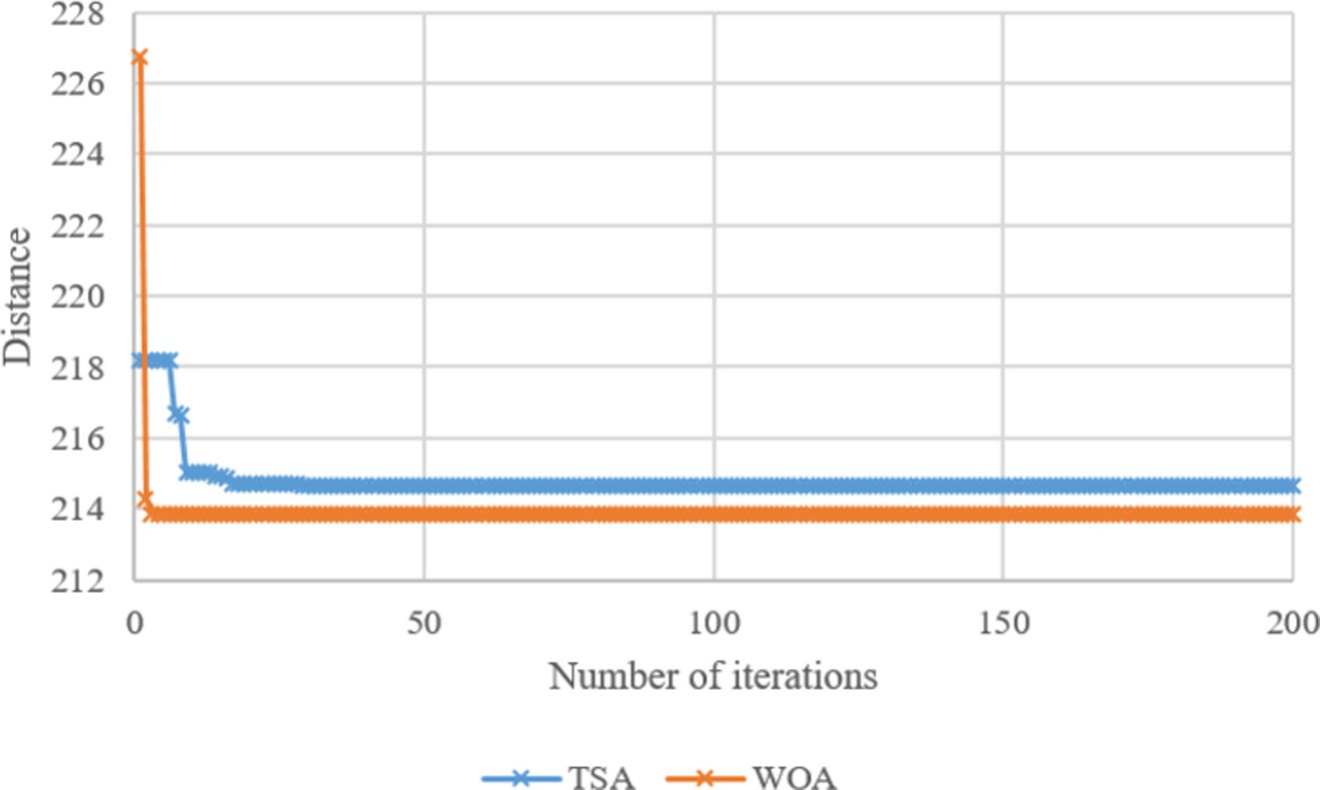
Search history of TSA and WOA for Case-*60*.

Search histories of TSA and WOA for Case-*10*, Case-*20*, Case-*30*, Case-*40*, Case-*50*, and Case-*60* are given in **Figs 8–13**, respectively. Since there are 66 cases in this study, the six cases mentioned above were chosen as a sample to show the convergence rates of TSA and WOA. In other words, the TSA and WOA results of each situation were combined and plotted. However, the search history of WA could not be provided because packaged software was used to achieve the WA results provided. When the search histories are examined, the TSA method has almost completed the optimization procedure before reaching 10% of the number of iterations, that is, before reaching the 20th iteration. Only in Case-*40*, TSA attained its final objective value before the 25th iteration (**Fig 11**). Therefore, it is clearly observed that TSA converged very fast. Moreover, **Figs 8–13** demonstrate that WOA converges more slowly than TSA, however WOA achieves almost its best results as quickly as 10 iterations. The reason is that WOA is effective in finding a starting objective value close to the final objective value compared to TSA. Although the objective value at the first iteration in WOA is more than the first objective value of TSA in Case-*60*, WOA performs almost the same as TSA (**Fig 13**). According to the results, while TSA has a faster convergence to the *OV*, WOA generally has better outputs because it usually starts from a better place to find the *OV*.

The performances of the methods are very close to each other. The reason for this is the following. Due to the low number of parameters in the study, both TSA, WA, and WOA have easily reached the solution. All methods achieved good results with each solution. Therefore, the standard deviations are almost similar for every method, and a closeness appears for the standard deviations (**Table 6**). That is to say, the maximum difference between the standard deviations is 0.58. Thus, it is observed that these three methods perform well because the values ​​are very close to each other, and the differences are very small. In addition, according to the results, these three methods show a very consistent behavior and find similar results each time. In other words, when the WA, TSA, and WOA results are compared, their standard deviations are not very high from each other, and *t*-test results also show that there is not much difference between the means. Thus, these three methods do not show a fluctuating performance compared to each other.

**Fig 14:**
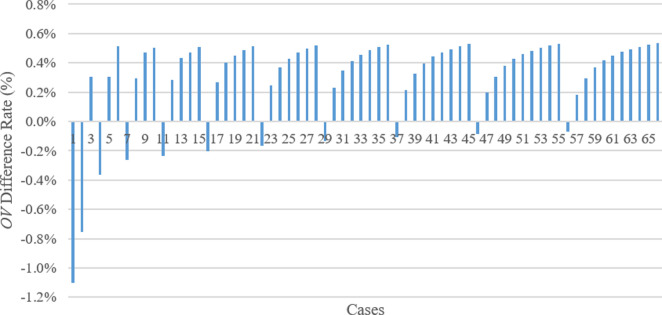
Comparison between WA and TSA in terms of *OV* difference rate.

Moreover, the *OV*s from the techniques were compared according to the results achieved (**S1 Table**). The best *OV* is highlighted in bold and dark blue, the second-best *OV* is shown in blue, and the third-best *OV* is seen in light blue color in **S1 Table**. When we compare WA and TSA, the following outcomes have been observed. Given minus percentage means TSA has less *OV* for the corresponding case, and thus TSA is better (**Fig 14**). TSA achieved better results or *OV*s than WA in 11 of 66 solutions and WA compared to TSA in 55 solutions. However, their difference is not more than 1.1% when the *OV* difference for a specific case is demonstrated as a percentage. Additionally, although the number of results is small where TSA’s *OV*s are lower than WA’s *OV*s, the difference percentage is relatively great and significant in such cases. For example, the TSA performed better in the cases such as the 1st, 2nd, 4th, 7th, 11th, 16th, 22nd, 29th, 37th, 46th, and 56th cases. In these cases, there is a particular situation that *P3* is zero. That is, TSA performs better when *P3* is zero. Accordingly, as the importance of *P3* increases, the differences between the *OV*s of WA and TSA increase. However, although applying the WA technique is partially accurate, it does not provide a significant performance advantage over TSA. Thus one can conclude that their performance is close to each other.

**Fig 15:**
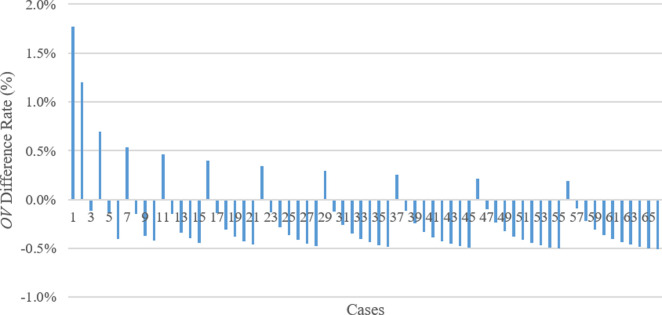
Comparison between TSA and WOA in terms of *OV* difference rate.

**Fig 16:**
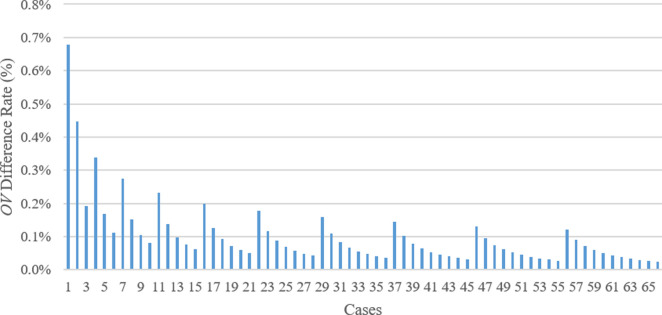
Comparison between WA and WOA in terms of *OV* difference rate.

In addition, **Fig 15** demonstrates the comparison of the results or *OV* of TSA and WOA techniques. In **Fig 15**, minus percentage means WOA creates less *OV* than TSA achieves for the corresponding case. Accordingly, in 55 of 66 solutions, WOA achieved better results or *OV*s compared to TSA. Therefore, TSA found better *OV*s compared to WOA in 11 solutions. However, since the percentage difference between the *OV*s of both methods is less than 1.8%. In the cases where TSA achieved less or better *OV*s compared to WOA’s outputs, the third parameter (*P3*) is zero. In fact, when the *P3* value increases, the difference rate between the TSA and WOA outputs increases. Additionally, TSA performed better in the initial cases, such as the 1st, 2nd, and 4th cases; that is, the *OV* difference rate is also higher (i.e., 1.77%, 1.20%, and 0.70%, respectively) compared to the other *OV* difference rates among TSA and WOA results. On the other hand, as the *OV*s of WA and WOA are compared (**Fig 16**), the difference rate between the WA and WOA results descends (the opposite tendency of the situations in **Figs 14** and **15**) when *P3* importance in the cases increases. Although the results of the WA technique are slightly better than the ones computed by WOA, the highest difference rate between the outputs of both methods is less than 0.7%. Thus the aftermath exhibits that performances of WA and WOA are similar.

## Conclusions

We produced a perception for the placement of an aid facility for immediate intervention during disasters by considering several spatial parameters. Furthermore, a test was conducted for understanding not only the influence of different parameters of the optimization problem but also the performance of WA, TSA, and WOA. WA has considerable drawbacks to process data as the data get larger in discrete network problems. It can achieve feasible solutions with comparably small size data, however big data challenge the method. Shortly, as the data size expands, WA performance decays in discrete problems. On the other hand, WA could handle the problem in this study with a continuous network as good as TSA and WOA did. This was proved by the *OV*s purchased. More than half of the *OV*s found by TSA in all cases (67%) were numerically greater than the ones computed by WA. This shows WA can be nearly flexible methodology in continuous networks. However, TSA is able to adapt to handle big data in both large discrete and continuous problems. Flexibility of TSA is noticeably a strength for an increase at the number of parameters in discrete networks.

Since WA is a special algorithm developed for the cases such as the problem in this study, WA’s performance of finding generally better outputs than the others could be predicted. In this study, the important point we were wondering about was how close the meta-heuristic methods are to WA. However, meta-heuristic techniques such as TSA and WOA came pretty close to WA performance, even sometimes providing better products. Such that the *OV* difference remains below 1% in all results with better WA *OV*s. Although WA usually delivers better *OV*s, algorithms such as TSA and WOA can still be recommended for the optimization problems like in this study because the differences in *OV*s are very small (less than 1%). The reason for our recommendation is as follows. Ultimately, algorithms such as WA are suitable for specific problems as proposed in this study, also applied successfully in the other works [49, 80]. Nevertheless, the effectiveness of ready-made algorithms such as WA becomes inapplicable when different parameters and different variables are integrated into them. On the other hand, the flexibility of meta-heuristic algorithms is so great that even if there are substantial changes in the problem structure and we affect many parameters, as in several studies [11, 14, 64], these methods can be easily applied. In addition, the performance of these methods is not affected or fluctuated much, which is compatible with the previous studies. Therefore, meta-heuristic methods such as TSA and WOA can be applied for the type of problems in this study as many researchers operate effectively [82, 83], and are pretty powerful to find the optimum solutions.

Further exploration and research can be considered for future analysis by integrating new parameters, such as regional distinctions where the airports are located specifically. Moreover, the increment value of the importance weights of the parameters can be varied to observe the possibility of change in optimal TFLEEs and *OV*s. Furthermore, by rerunning WA compared to running a couple of contemporary algorithms, the location shifts or differences between new and old TFLEEs can be revealed. Thus, more discussion could be made regarding the performance of the algorithms.

## Supporting information

S1 Table Optimum locations and *s*according to the cases and methods. (DOCX)
