# Supplementary material for: Weiszfeld, tree-seed, and whale optimization algorithms comparison via locating transportation facilities with weightings considering the vulnerability and uncertainty
Source: PLoS One. 2022 Jun 14;17(6):e0269808. doi: 10.1371/journal.pone.0269808 (PMC9197024; doi:10.1371/journal.pone.0269808)
Supplement: S1 Table — (DOCX) [file pone.0269808.s001.docx]

**S1 Table. Optimum Locations and** $\boldsymbol{OV}$**s According to the Cases and Methods.**

| **Cases** | ***P1* (%)** | ***P2* (%)** | ***P3* (%)** | **Optimum Location (WA)** | | **Optimum Location (TSA)** | | **Optimum Location (WOA)** | | $\boldsymbol{OV}$ **(WA)** | $\boldsymbol{OV}$ **(TSA)** | $\boldsymbol{OV}$ **(WOA)** |
| --- | --- | --- | --- | --- | --- | --- | --- | --- | --- | --- | --- | --- |
|  |  |  |  | **Latitude** | **Longitude** | **Latitude** | **Longitude** | **Latitude** | **Longitude** |  |  |  |
| Case-*1* | 100 | 0 | 0 | 40.905399 | 29.316898 | **40.769786** | **29.262619** | 40.906035 | 29.316988 | 18.1261 | **17.9264** | 18.2499 |
| Case-*2* | 90 | 10 | 0 | 40.499711 | 29.571756 | **40.372669** | **29.572499** | 40.499464 | 29.572001 | 28.0370 | **27.8256** | 28.1628 |
| Case-*3* | 90 | 0 | 10 | **39.566385** | **31.830446** | 39.480860 | 31.805733 | 39.569611 | 31.834019 | **62.6409** | 62.8311 | 62.7611 |
| Case-*4* | 80 | 20 | 0 | 39.783554 | 30.405108 | **39.677958** | **30.341677** | 39.783840 | 30.405444 | 37.1471 | **37.0124** | 37.2728 |
| Case-*5* | 80 | 10 | 10 | **39.490566** | **32.249405** | 39.404485 | 32.239093 | 39.493924 | 32.252807 | **70.4638** | 70.6791 | 70.5830 |
| Case-*6* | 80 | 0 | 20 | **39.587379** | **33.190477** | 39.529963 | 33.249491 | 39.590123 | 33.191254 | **100.8272** | 101.3469 | 100.9405 |
| Case-*7* | 70 | 30 | 0 | 39.516424 | 30.822652 | **39.327880** | **30.878088** | 39.517128 | 30.823292 | 45.7323 | **45.6113** | 45.8579 |
| Case-*8* | 70 | 20 | 10 | **39.444712** | **32.560786** | 39.360081 | 32.557512 | 39.448087 | 32.563597 | **78.1182** | 78.3497 | 78.2366 |
| Case-*9* | 70 | 10 | 20 | **39.497825** | **33.373740** | 39.443847 | 33.441614 | 39.500111 | 33.374702 | **108.1702** | 108.6832 | 108.2837 |
| Case-*10* | 70 | 0 | 30 | **39.434107** | **34.137008** | 39.404485 | 34.216509 | 39.435826 | 34.137776 | **137.1035** | 137.7944 | 137.2135 |
| Case-*11* | 60 | 40 | 0 | 39.314015 | 31.294665 | **39.152154** | **31.316797** | 39.315275 | 31.296090 | 54.0150 | **53.8877** | 54.1401 |
| Case-*12* | 60 | 30 | 10 | **39.401557** | **32.785598** | 39.314015 | 32.793733 | 39.404714 | 32.787664 | **85.6587** | 85.9010 | 85.7767 |
| Case-*13* | 60 | 20 | 20 | **39.420482** | **33.572730** | 39.368446 | 33.646382 | 39.422476 | 33.573852 | **115.4377** | 115.9410 | 115.5512 |
| Case-*14* | 60 | 10 | 30 | **39.377292** | **34.269789** | 39.347476 | 34.338942 | 39.378874 | 34.270409 | **144.1543** | 144.8395 | 144.2643 |
| Case-*15* | 60 | 0 | 40 | **39.379395** | **34.617488** | 39.368388 | 34.676478 | 39.380822 | 34.618077 | **172.3056** | 173.1822 | 172.4128 |
| Case-*16* | 50 | 50 | 0 | 39.198776 | 31.732232 | **39.057502** | **31.730960** | 39.200455 | 31.734225 | 62.0462 | **61.9212** | 62.1707 |
| Case-*17* | 50 | 40 | 10 | **39.350237** | **32.969936** | 39.264397 | 32.985797 | 39.353033 | 32.971474 | **93.1158** | 93.3636 | 93.2335 |
| Case-*18* | 50 | 30 | 20 | **39.354150** | **33.772735** | 39.303071 | 33.848048 | 39.355955 | 33.773878 | **122.6333** | 123.1260 | 122.7467 |
| Case-*19* | 50 | 20 | 30 | **39.327817** | **34.378975** | 39.298316 | 34.436060 | 39.329370 | 34.379524 | **151.1672** | 151.8496 | 151.2774 |
| Case-*20* | 50 | 10 | 40 | **39.347109** | **34.680021** | 39.336360 | 34.734602 | 39.348450 | 34.680624 | **179.2472** | 180.1273 | 179.3546 |
| Case-*21* | 50 | 0 | 50 | **39.377584** | **34.900478** | 39.374691 | 34.958660 | 39.378702 | 34.901023 | **207.1045** | 208.1718 | 207.2092 |
| Case-*22* | 40 | 60 | 0 | 39.128520 | 32.116590 | **38.997513** | **32.104365** | 39.130496 | 32.118681 | 69.8704 | **69.7554** | 69.9941 |
| Case-*23* | 40 | 50 | 10 | **39.294443** | **33.146354** | 39.211856 | 33.173032 | 39.296826 | 33.147707 | **100.5011** | 100.7506 | 100.6188 |
| Case-*24* | 40 | 40 | 20 | **39.295841** | **33.961805** | 39.247494 | 34.031671 | 39.297456 | 33.962888 | **129.7636** | 130.2463 | 129.8770 |
| Case-*25* | 40 | 30 | 30 | **39.285751** | **34.468410** | 39.256891 | 34.517488 | 39.287200 | 34.468943 | **158.1504** | 158.8318 | 158.2606 |
| Case-*26* | 40 | 20 | 40 | **39.318994** | **34.737893** | 39.307082 | 34.788921 | 39.320260 | 34.738510 | **186.1724** | 187.0543 | 186.2798 |
| Case-*27* | 40 | 10 | 50 | **39.352586** | **34.945591** | 39.347132 | 35.001897 | 39.353606 | 34.946075 | **213.9944** | 215.0657 | 214.0992 |
| Case-*28* | 40 | 0 | 60 | **39.376381** | **35.099862** | 39.375780 | 35.154333 | 39.377269 | 35.100256 | **241.6854** | 242.9464 | 241.7877 |
| Case-*29* | 30 | 70 | 0 | 39.082975 | 32.419127 | **38.956546** | **32.403232** | 39.085175 | 32.421032 | 77.5357 | **77.4320** | 77.6587 |
| Case-*30* | 30 | 60 | 10 | **39.240155** | **33.329589** | 39.163270 | 33.364510 | 39.242223 | 33.330958 | **107.8184** | 108.0669 | 107.9358 |
| Case-*31* | 30 | 50 | 20 | **39.243272** | **34.130434** | 39.194782 | 34.198314 | 39.244801 | 34.131326 | **136.8375** | 137.3114 | 136.9507 |
| Case-*32* | 30 | 40 | 30 | **39.250806** | **34.543204** | 39.222914 | 34.586527 | 39.252193 | 34.543780 | **165.1099** | 165.7918 | 165.2201 |
| Case-*33* | 30 | 30 | 40 | **39.294082** | **34.792507** | 39.280554 | 34.844521 | 39.295279 | 34.793109 | **193.0831** | 193.9676 | 193.1905 |
| Case-*34* | 30 | 20 | 50 | **39.329141** | **34.988196** | 39.321119 | 35.043610 | 39.330115 | 34.988661 | **220.8735** | 221.9488 | 220.9783 |
| Case-*35* | 30 | 10 | 60 | **39.353790** | **35.131517** | 39.350054 | 35.183326 | 39.354637 | 35.131845 | **248.5444** | 249.8077 | 248.6468 |
| Case-*36* | 30 | 0 | 70 | **39.373419** | **35.236445** | 39.374977 | 35.285348 | 39.374233 | 35.236739 | **276.1388** | 277.5880 | 276.2389 |
| Case-*37* | 20 | 80 | 0 | 39.052007 | 32.657387 | **38.930190** | **32.636724** | 39.054236 | 32.659157 | 85.0860 | **84.9940** | 85.2086 |
| Case-*38* | 20 | 70 | 10 | **39.190468** | **33.523527** | 39.118980 | 33.563874 | 39.192261 | 33.524908 | **115.0686** | 115.3141 | 115.1858 |
| Case-*39* | 20 | 60 | 20 | **39.195636** | **34.271839** | 39.147513 | 34.332460 | 39.197074 | 34.272540 | **143.8645** | 144.3323 | 143.9777 |
| Case-*40* | 20 | 50 | 30 | **39.222198** | **34.608420** | 39.195642 | 34.649380 | 39.223545 | 34.609024 | **172.0502** | 172.7338 | 172.1605 |
| Case-*41* | 20 | 40 | 40 | **39.271553** | **34.844610** | 39.256834 | 34.895337 | 39.272647 | 34.845226 | **199.9806** | 200.8677 | 200.0880 |
| Case-*42* | 20 | 30 | 50 | **39.306967** | **35.028386** | 39.297915 | 35.080331 | 39.307884 | 35.028832 | **227.7426** | 228.8220 | 227.8474 |
| Case-*43* | 20 | 20 | 60 | **39.332246** | **35.161177** | 39.326964 | 35.210903 | 39.333094 | 35.161500 | **255.3959** | 256.6649 | 255.4984 |
| Case-*44* | 20 | 10 | 70 | **39.353005** | **35.258483** | 39.352689 | 35.305970 | 39.353778 | 35.258731 | **282.9795** | 284.4366 | 283.0797 |
| Case-*45* | 20 | 0 | 80 | **39.371591** | **35.333127** | 39.374347 | 35.378646 | 39.372285 | 35.333366 | **310.5159** | 312.1607 | 310.6139 |
| Case-*46* | 10 | 90 | 0 | 39.028842 | 32.861277 | **38.912142** | **32.841644** | 39.030974 | 32.862967 | 92.5488 | **92.4687** | 92.6709 |
| Case-*47* | 10 | 80 | 10 | **39.145697** | **33.724813** | 39.078071 | 33.767496 | 39.147284 | 33.726238 | **122.2528** | 122.4941 | 122.3697 |
| Case-*48* | 10 | 70 | 20 | **39.153701** | **34.383633** | 39.106375 | 34.427034 | 39.155076 | 34.384158 | **150.8549** | 151.3191 | 150.9681 |
| Case-*49* | 10 | 60 | 30 | **39.198753** | **34.668161** | 39.172895 | 34.707712 | 39.199996 | 34.668844 | **178.9744** | 179.6610 | 179.0846 |
| Case-*50* | 10 | 50 | 40 | **39.250732** | **34.894501** | 39.233686 | 34.945746 | 39.251734 | 34.895055 | **206.8661** | 207.7557 | 206.9735 |
| Case-*51* | 10 | 40 | 50 | **39.285865** | **35.066226** | 39.273965 | 35.115255 | 39.286742 | 35.066639 | **234.6026** | 235.6862 | 234.7075 |
| Case-*52* | 10 | 30 | 60 | **39.311660** | **35.188959** | 39.304618 | 35.236037 | 39.312468 | 35.189273 | **262.2404** | 263.5154 | 262.3430 |
| Case-*53* | 10 | 20 | 70 | **39.333472** | **35.279141** | 39.330459 | 35.324438 | 39.334183 | 35.279361 | **289.8147** | 291.2795 | 289.9150 |
| Case-*54* | 10 | 10 | 80 | **39.353257** | **35.348878** | 39.355210 | 35.392520 | 39.353950 | 35.349116 | **317.3454** | 318.9992 | 317.4434 |
| Case-*55* | 10 | 0 | 90 | **39.371517** | **35.405377** | 39.378530 | 35.449016 | 39.372113 | 35.405597 | **344.8442** | 346.6846 | 344.9401 |
| Case-*56* | 0 | 100 | 0 | 39.008640 | 33.051322 | **38.900511** | **33.037110** | 39.010634 | 33.052959 | 99.9382 | **99.8695** | 100.0599 |
| Case-*57* | 0 | 90 | 10 | **39.104736** | **33.924535** | 39.041974 | 33.974103 | 39.106203 | 33.925909 | **129.3738** | 129.6111 | 129.4904 |
| Case-*58* | 0 | 80 | 20 | **39.118974** | **34.469383** | 39.072971 | 34.502616 | 39.120298 | 34.469888 | **157.8177** | 158.2811 | 157.9309 |
| Case-*59* | 0 | 70 | 30 | **39.179112** | **34.725222** | 39.154217 | 34.764355 | 39.180286 | 34.725891 | **185.8843** | 186.5722 | 185.9945 |
| Case-*60* | 0 | 60 | 40 | **39.231136** | **34.942186** | 39.212429 | 34.990210 | 39.232082 | 34.942740 | **213.7405** | 214.6325 | 213.8480 |
| Case-*61* | 0 | 50 | 50 | **39.265680** | **35.101756** | 39.252193 | 35.150070 | 39.266517 | 35.102131 | **241.4542** | 242.5418 | 241.5592 |
| Case-*62* | 0 | 40 | 60 | **39.291962** | **35.214938** | 39.282846 | 35.259761 | 39.292758 | 35.215210 | **269.0785** | 270.3592 | 269.1811 |
| Case-*63* | 0 | 30 | 70 | **39.314765** | **35.298494** | 39.310806 | 35.341218 | 39.315504 | 35.298723 | **296.6448** | 298.1171 | 296.7451 |
| Case-*64* | 0 | 20 | 80 | **39.335678** | **35.363696** | 39.336933 | 35.405643 | 39.336360 | 35.363883 | **324.1706** | 325.8331 | 324.2687 |
| Case-*65* | 0 | 10 | 90 | **39.355010** | **35.417014** | 39.359794 | 35.459657 | 39.355612 | 35.417224 | **351.6665** | 353.5170 | 351.7624 |
| Case-*66* | 0 | 0 | 100 | **39.372795** | **35.462103** | 39.382770 | 35.505333 | 39.373373 | 35.462271 | **379.1392** | 381.1762 | 379.2330 |
